
JOURNAL INFORMATION
==============================
NLM Title Abbreviation: ScientificWorldJournal
Journal ID: ScientificWorldJournal
NLM Journal ID: 101131163
Journal ID: TSWJ

The Scientific World Journal

ISSN: 2356-6140
EISSN: 1537-744X
Publisher: Wiley

ARTICLE INFORMATION
==============================
PMCID: PMC6009571
PMID: 29973850
DOI: 10.1100/tsw.2002.173
Article ID: 539654
Article version: 1
Subjects: Founding Editorial

Founding Editorial — The Pharmaceutical Sciences

Chamberlain Joseph *
Milton Keynes, UK
*Joseph Chamberlain: spiresgate@aol.com
Publication date: 2002
Electronic publication date: 2002 Mar 27
Volume: 2
First page: 842
Last page: 843
Received 2002 Feb 15; Accepted 2002 Feb 15
Copyright: Copyright © 2002 Joseph Chamberlain.
Copyright year: 2002
License: This is an open access article distributed under the Creative Commons Attribution License, which permits unrestricted use, distribution, and reproduction in any medium, provided the original work is properly cited.
License URL: https://creativecommons.org/licenses/by/3.0/

Keywords: electronic publishing, intelligent documents, animations

==============================
Founding Editorial
*TheScientificWorldJOURNAL* (2002) 2, 842–843
ISSN 1537-744X; DOI 10.1100/tsw.2002.173

### Founding Editorial — The Pharmaceutical Sciences

Joseph Chamberlain
*Milton Keynes*

*Received February 15, 2002; Accepted February 15, 2002; Published March 27, 2002*

**KEY WORDS:** electronic publishing, intelligent documents, animations

**DOMAINS:** pharmaceutical sciences, pharmacokinetics, molecular modeling

Pharmaceutical science may be defined as all those activities concerned with the discovery, detection, evaluation, safety, development, and formulation of drugs and medicines. Each of these activities in turn will encompass many disciplines in the scientific world, such as chemistry, biochemistry, pharmacology, biology, toxicology, analytical chemistry, materials science, molecular modelling, drug metabolism, pharmacokinetics, computer simulations, and clinical sciences. A glance at the reference list for any research paper in the pharmaceutical sciences will reveal that even for specialised pieces of research, reference will be made to journals in the disparate fields covered by the pharmaceutical sciences. It is this wide range of disciplines that makes a concept such as *TheScientificWorldJOURNAL* so attractive for the pharmaceutical scientist; relevant articles from many different sources can be selected and directed to the researcher, without the need to wade through masses of unrelated journal articles.

However, electronic publishing does not only improve the timing and distribution of conventionally published articles; it has tremendous potential for bringing to the reader facets that are not easily brought with the normal printed page, such as intelligent documents, animations, and full colour. This is not the place to promote the advantages of electronic publishing in general, but it is worth pointing out that some aspects of the pharmaceutical sciences are particularly amenable to improvement in this way. For example, pharmacokinetic reports often need to be limited to summaries of the relevant parameters thought necessary to convey the properties of a particular drug; however, this involves an interpretation by the author, an interpretation which may be open to a different interpretation by a third party with perhaps particular knowledge of the experimental situation. With an intelligent document, the data underlying the summary report can be included in the electronic publication. The use of colour and animation is particularly useful for molecular modelling where the demonstration of three-dimensional modelling cannot otherwise be demonstrated without special viewers or optical gymnastics that not all readers can command.

Nevertheless, if electronic publishing — and particularly on-line electronic publishing — is the future of dissemination of scientific research, and if the science community is to take full

Email: Spiresgate@aol.com
©2002 with author.

advantage of the many claims for its superiority over the printed version, then there is, in my opinion, a final barrier yet to be overcome. Nowadays, the familiarity of most laboratory scientists with keyboard techniques means that preparation of research papers for publication no longer relies on the services of a typist. Indeed computer packages enabling the laboratory worker to produce research scripts of publishable standard (according to the salesman, that is) may too often have deluded the researcher into believing the work itself was of publishable standard. By moving the publication process another step further towards communications that would not otherwise be possible without the electronic revolution, the dependency on a typist may now have been replaced by a dependency on personnel with considerable computer skills. These skills may include not only the use of graphics, database, and spreadsheet packages, but the potential to integrate them into web pages. Here again the immediate impact might be a case of 'great graphics, shame about the science!'

The other side of the coin is that there may be situations where researchers carrying out perfectly good science may lack the opportunity of optimal presentation for lack of the necessary skill with the computing tools. The role of *TheScientificWorldJOURNAL* in the new climate is twofold.

Firstly, the scientific standards of the work presented must be upheld. To this end, an Editorial Board has been created from respected workers in the full range of the pharmaceutical sciences. The work of the individual Board Members will be well-known in their respective fields and I have personal experience of their dependability and fairness. This is an excellent start.

Secondly, TheScientificWorld must use its pre-emininent position in electronic publishing to help those authors with limited computer resources with regard to presentation techniques. I am not yet sure how this may be done, but obviously there must be close collaboration between the computer wizards of TheScientificWorld and the research workers. Instructions and guidelines for electronic publishing will need to be followed very carefully. We must guard, of course, against elaborate use of colour and graphics for the sake of it, but there will be situations where the research results could not be successfully presented by conventional means.

I look forward to this venture with great excitement and interest. I hope the diverse community of pharmaceutical scientists will take up the challenge of the revolution in publishing both by bringing their work to the notice of a wider community via the concept of *TheScientificWorldJOURNAL* and by full use of the facilities only available in the electronic form.

**This article should be referenced as follows:**

Chamberlain, J. (2002) Founding editorial — The Pharmaceutical Sciences. *TheScientificWorldJOURNAL* **2**, 842–843.
